# Removal of an implanted central venous catheter from neutropenic patients admitted to the ICU due to sepsis from any source

**DOI:** 10.1186/cc14155

**Published:** 2015-03-16

**Authors:** B Civantos Martin, I Pozuelo Echegaray, C Guallar Espallargas, A Robles Caballero, C Briones, P Extremera Navas, P Millan Estañ, A Garcia de Lorenzo y Mateos

**Affiliations:** 1 Hospital Universitario La Paz, Madrid, Spain

## Introduction

Long experience in the treatment of neutropenic patients admitted to the ICU has taught us the importance of removing the permanent central venous catheter when infection is suspected, because of the great mortality associated. The problem usually comes when the origin of sepsis is not clear and we assume that mortality is not easy to avoid. It is important to know what happens to neutropenic patients admitted to the ICU because of sepsis from any source, in whom catheter infection cannot be excluded.

## Methods

A retrospective, cohort, descriptive study was carried out between January 2013 and November 2014. Epidemiology data were collected from all neutropenic patients admitted to the ICU who came from hemato-oncology services and had an implanted central venous catheter. Microbiology results and data related to the catheter removal were described.

## Results

A total of 15 patients were included, mean age was 53 years old and 66% were male. The implanted catheter was removed in 80% of patients. Platelet transfusion was needed in 100% of patients before catheter removal and no complication was observed during catheter removal or in the insertion of a new one. In 53% of patients, catheter infection was confirmed *a posteriori*.

## Conclusion

Removal of an implanted central venous catheter from neutropenic patients admitted to the ICU due to sepsis from any source can be beneficial for this kind of patient as it was found that in more than 50% of patients catheter infection was confirmed *a posteriori*.

